# Contracting non-state providers for universal health coverage: learnings from Africa, Asia, and Eastern Europe

**DOI:** 10.1186/s12939-018-0846-5

**Published:** 2018-10-05

**Authors:** Krishna D. Rao, Ligia Paina, Marie-Gloriose Ingabire, Zubin C. Shroff

**Affiliations:** 1 Department of International Health, Johns Hopkins University, Suite E-8148 315 N. Wolfe Street, Baltimore, MD 21215 USA; 2 International Development Research Centre, Ottawa, Canada; 3 Alliance for Health Policy and Systems Research, WHO, Geneva, Switzerland

**Keywords:** Non-state providers, Universal health coverage, Contracting

## Abstract

**Background:**

Formal engagement with non-state providers (NSP) is an important strategy in many low-and-middle-income countries for extending coverage of publicly financed health services. The series of country studies reviewed in this paper - from Afghanistan, Bangladesh, Bosnia & Herzegovina, Ghana, South Africa, Tanzania and Uganda – provide a unique opportunity to understand the dynamics of NSP engagement in different contexts.

**Methods:**

A standard template was developed and used to summarize the main findings from the country studies. The summaries were then organized according to emergent themes and a narrative built around these themes.

**Results:**

Governments contracted NSPs for a variety of reasons – limited public sector capacity, inability of public sector services to reach certain populations or geographic areas, and the widespread presence of NSPs in the health sector. Underlying these reasons was a recognition that purchasing services from NSPs was necessary to increase coverage of health services. Yet, institutional NSPs faced many service delivery challenges. Like the public sector, institutional NSPs faced challenges in recruiting and retaining health workers, and ensuring service quality. Properly managing relationships between all actors involved was critical to contracting success and the role of NSPs as strategic partners in achieving national health goals. Further, the relationship between the central and lower administrative levels in contract management, as well as government stewardship capacity for monitoring contractual performance were vital for NSP performance.

**Conclusion:**

For countries with a sizeable NSP sector, making full use of the available human and other resources by contracting NSPs and appropriately managing them, offers an important way for expanding coverage of publicly financed health services and moving towards universal health coverage.

## Background

The health workforce of many low-and-middle-income countries (LMICs) is characterized by a mix of state and non-state providers (NSPs). NSPs or private sector health care providers are a heterogeneous group. They comprise for-profit and not-for-profit entities that include corporate hospitals, mission hospitals, non-government organizations, individual practices, or private pharmacies. NSPs can practice allopathic, traditional or faith based systems of medicine. They can be formally trained or not; in several countries unqualified NSPs comprise a large portion of the health workforce [1]. Participation of NSPs in service delivery can vary substantially across countries. In Nigeria, for instance, close to 80% of outpatient visits are provided by them while it is 29% in Malawi. Similarly, in India they treat around 62% of hospital admissions, while the corresponding figure for Thailand is just 10% [2].

Formal engagement with NSPs is an important component of health system reforms in many LMICs [3]. This engagement has been motivated by various reasons – to extend coverage of publicly financed health services by taking advantage of all available resources in the health sector, establishing quality standards for NSP provided care, and increasing accountability among them. In the context of universal coverage, engaging NSPs can extend coverage of publicly financed health services in underserved geographic areas and hard to reach populations. Countries have engaged NSPs through two principal mechanisms – formal government contracts to deliver health services, or through strategic purchasing of specific services [4]. In either case, from a health financing perspective, public funds are used to purchase services from NSPs.

In 2014, The Alliance for Health Policy and Systems Research, in collaboration with the International Development Research Centre (IDRC), Canada and the Rockefeller Foundation, launched a programme of research to examine the role of NSPs in strengthening health systems towards the achievement of universal health coverage in a range of LMICs. Details on the programme of research, as well as an overview of each of the papers, is provided in the introductory paper by Shroff et al. [5]. Studies from each of the seven countries included in this article collection, namely Afghanistan, Bangladesh, Bosnia & Herzegovina, Ghana, South Africa, Tanzania and Uganda – provide a unique opportunity to understand the ways in which NSPs have been engaged in different contexts with the common purpose of moving towards universal health coverage. In this paper, we report on the main learnings on the process of engaging NSPs from the experience of these seven countries. All the country studies, with one exception, focused on contracting as a means of engaging NSPs. The exception was the Bosnia & Herzegovina study which focused on adoption of quality standards among NSPs. The aim of these country studies was to understand the dynamics between context, policies, and actors that influence NSP contracting and performance, a relatively understudied area which this research program sought to fill.

Contracting NSPs takes place in the context of a formal understanding (“contract”) between the government and NSP that requires the NSP to provide a specific set of services on behalf of the government for some agreed compensation. There are several different mechanisms through which NSPs can be contracted. In management contracts, NSPs provide services within existing government establishments. On the other hand, in service delivery contracts, contracted NSPs provide services using their own infrastructure and resources [6]. For example, in India the government undertakes large scale contracting of individual doctors to work in existing public sector health facilities [7], while in Afghanistan, contracted NGOs provide health services in entire provinces using their own health facilities [8].

Contracting fundamentally changes the role of government in the health sector. In many LMICs, governments see themselves as being responsible for financing as well as provision of health services to their citizens. Contracting changes this by placing the responsibility for service delivery on contracted NSPs, while the role of government moves to financing health services and providing stewardship over contracted providers. The stewardship role requires governments to monitor contract performance – this can be done either by the government or more objectively by a third party [9].

Evidence on the effectiveness of contracting on service use and health outcomes is mixed. A recent Cochrane review found that while there was evidence that contracting reduces out-of-pocket spending on curative care, there was little or no difference in the use of preventive health services (e.g. antenatal care, immunization, contraception), and health outcomes (infant mortality, diarrhea) [10]. Another review also found that there is weak evidence that contracting increased access and utilization of health services [3]. These reviews cite the need for better evidence on the effects of contracting. Other reviews, however, have found that contracting NSPs is associated with increased coverage of health services and reduced socioeconomic inequities in service use [6]. Importantly, the policy of contracting NSPs is shaped as much by necessity and political considerations as it is by considerations of effectiveness [11]. Such issues are particularly salient because, as the country studies included in this special issue indicate, contracting NSPs continues to be an important strategy for LMICs to move towards universal coverage.

## Methods

Project reports prepared by the seven country teams, and related papers included in this article collection, were the chief sources of information for this review paper. These country studies broadly reported on the role of NSPs in strengthening health systems towards the achievement of universal health coverage. Characteristics of these country studies are described in Table 1. A range of NSPs, such as national and international non-governmental organizations (NGO), for-profit private providers and faith based non-profit providers, were represented in the study countries. The NSPs studied were largely involved in providing rural primary care services, except in Bangladesh and Bosnia & Herzegovina, where they were focused on delivering urban health services.

**Table 1:** Characteristics of the country studies

	Country	Authors	Type of NSP	Service provided by NSP	Focus of Country Study
1	Afghanistan	Salehi, Saljuqui, Akseer, Rao, & Coe.	National and international NGOs	Rural and urban primary health care and hospital services	Factors that influenced NSPs performance in implementing basic package of health services.
2	Bangladesh	Islam, Hossain, Bashar, Khan, Sikder, Yusuf, & Adams.	Mostly national NGOs	Urban primary health care services	Factors that influenced the evolution and implementation of contracting-out health services.
3	Bosnia & Herzegovina	Rakic, Novakovic, Stevic, & Niskanovic.	For-profit private providers	Pharmacy, dental and specialist care.	Introduction of safety and quality standards for private health care providers.
4	Ghana	Grieve, & Olivier.	Faith-based non-profit providers	Rural primary health care	Mapping the development of the faith-based non-profit sector
5	South Africa	Mureithi, Burnett, Bertscher & English.	For-profit general physicians	Rural and urban primary health care.	Assessment of three general practitioner contracting models.
6	Tanzania	Maluka, Chitama, Dungumaro, Masawe, Rao, & Shroff.	Faith-based non-profit providers	Rural primary health care services	Design and implementation of service agreements between local governments and NSPs.
7	Uganda	Ssennyonjo, Namakula, Kasyaba, Orach, Paina, Bennett, Ssengooba.	Faith-based non-profit providers	Rural primary health care services	Evolution of government resource contributions to, and partnerships with, NSPs.

The country studies were reviewed by the authors to identify emergent themes for inclusion in this paper. A standard template was developed and was used to summarize the main findings from the country studies. The summaries were then organized according to emergent themes and a narrative built around these themes. In the final stage, the authors of the country studies were asked to review the draft manuscript to see if it agreed with the messages that were elicited from their studies. The draft manuscript was revised based on feedback from the study authors. No additional human subjects were contacted for this study beyond the country papers, hence no additional ethical review was needed. However, the individual country studies had completed ethical review as outlined in the country articles.

The lessons from the seven country studies have been organized into the following sections, based on the emergent themes: the policy and health systems context within which contracting takes place, how contracting performance is affected by the government ownership of the process, the challenges that NSPs face in service delivery, monitoring contract performance, and the relationships embodied in the contracting process and its implications. The structure of the paper follows these themes.

## Results

### Non-state providers are typically contracted to complement public sector services

Governments contracted NSPs for a variety of reasons related to the particular context of their health system. In Afghanistan, at the end of decades of civil war in 2002, the country was left with a broken health care system, few human resources for health, dependency on external donors to finance health services, and virtually no government capacity to deliver health services [12]. It was natural, therefore, for the government and development partners to look towards local and international NSPs for delivering health services in these challenging circumstances. Through service delivery contracts, the overwhelming majority of health services in the 34 provinces in Afghanistan were contracted out to NSPs to deliver a basic package of health services. The Ministry of Public Health, through a specially established grants management unit, took on the role of managing the contracting process, and monitoring performance with the help of third party evaluators.

In many ways, Afghanistan is an atypical case because of the unique circumstances in which contracting was introduced. Bangladesh, and Tanzania represent more typical examples of contracting in LMICs. Here NSPs were contracted to increase coverage of publicly financed health services in areas where the existing public sector health system was weak. Bangladesh has historically had a strong NSP presence in health, exemplified by large scale homegrown NGOs such as BRAC, which made contracting them a viable option for delivering health services [13]. In particular, the lack of local government capacity to deliver basic health services to marginalized populations in urban areas motivated the engagement of NSPs [13]. Funding from a consortium of international donors to the Ministry of Local Government through the Urban Primary Health Care project in 1998 enabled urban local governments to contract NSPs to deliver basic health services.

In Tanzania the concerns that motivated contracting were related to the large geographic inequalities in health [14]. Rapid population and income growth, along with rapid urbanization, had resulted in substantial regional differences in health and use of health services. In particular, government health services were unable to adequately penetrate rural and remote areas of the country. To address these challenges the government looked towards contracting faith-based NSPs which have historically had a substantial presence in Tanzania’s health sector. Another advantage offered by formal contracts with NSPs in Tanzania, is that after public funds became available to the NSPs through contracting, it reduced their need to raise funds through user fees to recover their costs [14].

Ghana and South Africa represent LMICs that have made (or are in the process of making) serious efforts at moving towards universal coverage through a national health insurance program. The Ghana National Health Insurance Scheme and South Africa’s proposed National Health Insurance draw on NSPs to deliver health services. In Ghana, the historical and widespread presence of not-for-profit faith-based providers made them natural allies to the public sector for achieving universal coverage. Faith-based NSPs claim to provide 35% to 40% of healthcare services in the country and are also essential providers of tertiary care in certain areas [15]. They are largely aligned with the Christian faith, and are networked under the umbrella of the Christian Health Association of Ghana (CHAG) [15]. Though autonomous, CHAG has historically had strong ties with the public sector in Ghana and is a recognized agency of the Ministry of Health. Moreover, health facilities under it are integrated with the public sector in terms of reporting and have been fast tracked for accreditation with the Ghana National Health Insurance Scheme [15].

South Africa’s publicly funded National Health Insurance program represents an important attempt at universal coverage. This insurance program, which is yet to be implemented, will eventually be established as a single-payer and single-purchaser model to strategically purchase health care services from a mix of private and public providers. However, findings from the South Africa study suggests that the insurance program’s potential will be constrained by the limited capacity of the public sector to deliver primary care services [16]. For example, around 82% of the population depends on public services, yet roughly half of the overall health expenditure in the country goes to the private sector [16]. One of the fundamental challenges is that human resources in the health sector are overwhelmingly present in the private sector. For instance, while there are approximately 93 doctors per 100,000 people in the private sector, there are 25 per 100,000 in the public sector [16]. To strengthen public sector capacity to deliver services, on which the majority of the country’s population depends, South Africa piloted the General Practitioner Contracting Initiative (GPCI), as part of a plan to reengineer primary health care in the country and address the structural imbalances in terms of funding and human resources in the public sector. Through the GPCI, general practitioners (doctors) in the private sector are contracted by government to work in public sector facilities for a portion of their time. Three models of contracting emerged – identified as the centralized-purchaser model, decentralized-purchaser model, and contracted-purchaser model. The latter two models were adaptations of the centralized-purchaser model. All these models derived funding from a single central source but had varying levels of involvement of national, provincial and district managers [16]. Emergence of these two models was strongly influenced by the health system context, such as, purchaser capacity to manage contracts, payments and recruitment processes.

Bosnia and Herzegovina presents a country at the relatively high end of the universal coverage spectrum [17]. The country has widespread coverage of health services and relies on NSPs, who are engaged through the national social health insurance program. Since 2010, this has included contracting for specialist services, a measure that was introduced to increase access to these services in rural areas. As a country where healthcare coverage is not as major a policy issue as in some of the other contexts in the country studies, Bosnia & Herzegovina’s challenge has been to ensure quality of health services provided by NSPs. Certification regulation of NSPs was introduced in 2009 by the Ministry of Health and Social Welfare to improve safety, trust, quality and environmental protection. An independent agency for certification was created to assess provider’s compliance in 2012 and found considerable variation in provider compliance, specifically by pharmacists, specialist practices and dentists. Many of them chose not to adopt the standards in spite of compliance being mandatory.

### Contracting success depends on the level of government ownership

One of the important concerns with contracting NSPs is the effect it has on the role of government in the health sector [18]. In Afghanistan, because donors exclusively financed contracting, a harmonious relationship between the government and international donors was necessary. A key feature of NSP contracting in Afghanistan was that, despite markedly diverging views in procurement and contracting practices, international donors agreed to leave the Ministry of Public Health as the unique, centralized contractor in the country [12]. The Ministry’s position as the central authority for all matters of NSP contracting together with clear guidelines for the contracted services, a bidding system through which NSPs were selected, and continuous performance monitoring (though expensive) have been important reasons for the success of this model.

In contrast, the study of Bangladesh’s Urban Primary Health Care Project, documents how NSP contracting can be affected by weak government ownership of the process. The Ministry of Local Government was chosen as the executing agency for contracting due to historical partnerships with multilateral donor agencies [13]. However, the Ministry of Local Government had limited experience with health service delivery and was only marginally involved in the operation of the project. The Ministry of Health, which had considerably more experience in delivery of health services, was also a partner in the project but had no direct contractual obligations, which notably reduced its involvement and interest. Altogether, weak capacity and low sense of ownership at the central government level affected the implementation of the contracting model in Bangladesh. A related challenge is political interference, which compromises good governance of contracts. The Bangladesh study reported on how areas to be serviced by NSPs through a bidding process were selected or dropped at times due to political considerations rather on the basis of need [13].

The Tanzania study demonstrates that, in a decentralized system, only having central leadership in NSP administration is inadequate if there is weak governance at lower administrative levels. In Tanzania, finances for contracting were in the hands of the Ministry of Health, which also provided technical, financial and operational oversight [14]. A cost sharing guideline, designed at the central level, aimed to define the services provided by NSPs and to standardize the prices at which they were supplied. Local governments were in turn responsible for contracting in their areas and paying providers. In theory, the contracts allowed for a balanced set of responsibilities assigned to different players. However, contracting in Tanzania suffered from a general disregard for some of the formal elements of the contracts. For one, the process of selecting and assigning NSPs was perceived to lack transparency and technical rigor. The central government’s role in monitoring contracts was considered inadequate, and elements such as cost sharing guidelines were not followed. Moreover, local governments lacked capacity to adequately implement NSP contracts. For instance, local governments were not capable of raising the necessary cost sharing funds to make the model sustainable even as resources from external institutions declined.

Perhaps a key feature of successful NSP models is their ability to play to the strengths of the institutional capacity of the actors involved, as well as, their flexibility to adapt. The South African study reports that initially, the General Practitioner Contracting Initiative to contract private doctors into the public sector was implemented through a centralized-purchaser model, in which the National Department of Health recruited, contracted and managed the doctors, while the local (municipal or provincial) departments of health were in charge of program monitoring [16]. Importantly in terms of evolution of the models, payment delays in the centralized-purchaser model, for example, led to the hiring of an external organization for managing processes. This in turn resulted in the emergence of the contracted-purchaser model, in which an external organization is contracted to support partners at the district-level in the hiring and payment of doctors. Concurrently, a decentralized-purchaser model emerged in which a provincial department of health acted as the contracting agency which pays doctors through its payroll, thereby incorporating the NSPs into the payroll of the province. These changes reflect an iterative process of adaptation to the institutional capacity of the actors involved which was highly influenced by context, actors and capacity throughout the system. Ultimately, the transformations reflected the issues with the central government’s capacity to directly manage contracted doctors across the country.

### Service delivery challenges remain even after contracting non-state providers

In keeping with global evidence on the effectiveness of contracting, the country case studies included in this review also present mixed evidence on the effect of contracting on health care use. Yet, in all of the countries studied, NSPs were critical to the delivery of health services, particularly when the public sector had limited capacity as in Afghanistan or in geographical areas where public sector health services were weak as in Tanzania, Ghana, and Bangladesh. Moreover, as shown in the South Africa study, contracting can strengthen coverage of public sector services by attracting private physicians into the public sector [16].

Yet, not all service delivery issues are solved by simply contracting out services. Institutional NSPs can face many of the challenges that the public sector faces in recruiting and retaining health workers in rural or other underserved areas. In Bangladesh, for example, the Urban Primary Health Care Project struggled to retain managers and health care providers due to better salaries offered by the public sector and salary ceilings on contracted NSPs [13]. Challenges in attracting health workers to underserved areas can also skew NSP services towards already well-served areas. The study from Afghanistan, which by many accounts has been successful in large scale contracting of health services, reports that NSPs there continue to be challenged in finding female health workers [12]. In many areas of the country, the lack of female health workers is a major deterrent to women using health services.

Financing contracts – in terms of adequately financing service delivery costs, and consistent fund disbursements - is another important constraint to their effectiveness in service delivery. In Bangladesh and Tanzania, fluctuations in donor and government funding were reported to have affected NSP performance substantially. In Tanzania, while the districts had the authority to get into contractual agreements with NSPs, they had little capacity to generate financial resources to partly finance the contracts. As a result, the districts were dependent on the central government’s resources which in turn was dependent on donor support. Disbursement delays by donors or the central government hence hampered effective implementation of the contractual agreements.

Not preparing realistic budgets can also affect service delivery. In Tanzania, poor forecasting and planning for patient load, resulted in NSPs complaining about having inadequate budgets to cover service delivery costs. Another example from Bangladesh was that of awarding the contract for services to the lowest bidder who cleared a technical screening rather than basing the award itself on technical, or a mix of technical and cost, criteria. This led NGOs to bid as low as possible, something that had an adverse effect on service quality since providing quality services would put the NGO at a financial loss [13].

### Appropriately monitoring contract performance is important for service quality

An important concern with contracting NSPs is ensuring that they provide quality services. In South Africa, for example, one reason for deciding to contract doctors into the public system was the recognition that it would be difficult for the government to monitor or enforce quality of care if services were delivered outside public sector facilities [16]. Countries have evolved various mechanisms for monitoring contract performance. In Afghanistan, where large scale contracting was implemented, a third party monitored NSP performance to inform the government on quality of care and other service delivery issues [12]. A similar idea was tried with the ‘contracted-purchaser’ model in South Africa where an independent ‘district support partner’ at the district level was hired to manage contract performance. Other models of monitoring include, where the government (central or local) directly monitored performance (e.g. ‘centralized-purchaser’ model in South Africa), or through autonomous agencies (e.g. Bosnia-Herzegovina), or jointly by various stakeholders (e.g. government, NSP) via hospital boards (e.g. in Tanzania).

An important issue in managing contracts is deciding on a centralized or decentralized approach to monitoring. The South Africa study presents an interesting case where at different stages of the GPCI, government and non-government entities were involved in monitoring (and managing) contract performance. In the centralized contracting model that was first tried, the government through district level officers was responsible for monitoring performance of contracted health workers. However, experience with the contracted-purchaser model showed that monitoring contracts is easier when an independent party (i.e. district support partner) was responsible than a centralized authority. However, decentralization may not always be effective. In Tanzania, for example, district level officers were responsible for monitoring NSP performance. However, the Tanzania study reported that district officers seldom made monitoring and supervisory visits, due to lack of adequate resources and capacity.

Ensuring that NSPs comply with quality standards requires government involvement. In Bosnia & Herzegovina, a certification regulation was introduced in 2009 by the Ministry of Health and Social Welfare to improve safety, trust, and quality [17]. An independent agency for certification was created in 2012 to assess provider’s compliance with this regulation. The study from Bosnia and Herzegovina found there was a heterogeneous response to certification. Most of the certified pharmacies and specialists underwent the process because they felt it would benefit their management and increase their professional confidence and safety. Dentists did not perceive any substantial advantage in certifying and therefore largely abstained from doing so. Largely, providers (except for pharmacists) highlighted the downsides (costs, time, disruption of service) and claimed that patients would not recognize any change due to the certification. These findings suggest that when government has low enforcement capacity, it is difficult to ensure and monitor quality of NSP services.

### Contracts shape relationships between actors

Every contract embodies a certain kind of relationship between the contracting parties. At one extreme is the classical contract in which the relationship is built around the responsibilities of each party stipulated in the contract. On the other hand, in relational contracts, the specific stipulations of a contract are subordinated to building the overall relationship between the contracting parties [19]. Both forms of contracting arrangements were reported in the country case studies, and in some cases, they evolved from one form to another. In South Africa, the initial contracting model, the centralized-purchaser, was a classical contracting arrangement with clearly laid out responsibilities [16]. However, these stipulations were difficult to enforce due to limited government capacity. This contracting mechanism evolved into the contracted-purchaser contract when the government outsourced most aspects of contract management to an external organization. The decentralized-purchaser contract on the other hand was more relational due to the decentralized management nature of the model in that the local purchaser had built trust with the contracted physicians over many years.

In other contexts, such as Ghana, engagements between NSPs and government were more relational. As the study from Ghana reports, CHAG has a long-standing, semi-formalized relationship (through a signed Memorandum of Understanding) with the government and worked collaboratively but autonomously along with the public sector [15]. Through this relatively informal arrangement, CHAG facilities receive some financial assistance from government, and CHAG providers submit reports to the public sector health information systems.

In Tanzania, the relationship between NSPs and government evolved from a relational one to that of a classical contract. As the Tanzania study reports, the Tanzanian government has a long history of providing subsidies to Faith-Based Organisations (FBOs) to serve areas without public health facilities [14]. In 1992, the Government negotiated formal agreements with FBOs and in 2007 a new type of operational contract known as the Service Agreement (SA) was introduced. This marked the end of the relationship that was mainly informal and that relied primarily on trust and flexibility to a formal system, backed up by solid legal frameworks. However, the limited capacity of the district government to pay the contracted NSPs in a timely manner because of their dependency on central government funds, has adversely affected contractual relationships between the government and the NSPs [14]. Moreover, weak capacity for monitoring of contracts is also likely to have created a de facto informal relationship between the government and NSPs.

Though several types of contractual relationships exist in the countries studied, it is unclear if one form was clearly preferable over others. Having a classical contract with formalized obligations has its advantages in terms of delineating boundaries of responsibility and benchmarks for monitoring performance. However, weak governance mechanisms can render such formality redundant. Contractual arrangements that embody relationship building avoid these difficulties and NSP performance is built on trust and partnership with the government. There are weak mechanisms of accountability embodied in such arrangements, especially when financial transactions are involved due to government financing.

## Discussion

The series of country studies reviewed in this paper examined the role of NSPs in strengthening health systems towards the achievement of universal health coverage. The countries represented in these studies are at various stages of achieving universal coverage and of economic development. Their experience provides key insights into the dynamics between context, policies, and actors that influence NSP engagement. All these studies focused on one form of engagement i.e. through formal contracts between the government and NSPs. Several common themes emerged from these country studies. Governments contracted NSPs for a variety of reasons related to the particular context of their health system. These include inadequate public sector capacity, inability of public sector services to reach certain populations or areas and the existing widespread presence of NSPs. Behind all these factors was a recognition that in health systems contexts where there was a substantial NSP presence, purchasing services from NSPs was necessary to increase coverage of publicly funded health services.

Contracting is almost always an iterative and evolutionary process. Mureithi et al. (2018) demonstrate the evolution of South Africa’s decentralized purchaser model and contracted purchaser model from the initial centralized purchaser model. While such pilots have enabled the incorporation of learning based on implementation experience, they also facilitated the development of new capacities that enabled provinces to experiment with different models and build on existing ones, as demonstrated by the adoption of the decentralized-purchaser model [16]. The Bangladesh example too demonstrates the importance of learning from each phase informing the implementation of subsequent phases; the project altered financial rules in an earlier phase of the project that were seen as too onerous and demanding of smaller NGOs [13].

Several actors are involved in the contracting process and managing these relationships well is critical. One set of relationships involves those between the NSP and government. NSPs can be treated as contractors who are subordinate to the government or they can be treated as strategic partners in achieving national health goals. Relational contracts as seen in Ghana’s case are conducive for building the types of strategic partnerships that have a long term vision of the relationship between government and NSPs. A second set of relationships has to do with negotiating the administrative space between central and local governments. In several of the countries studied, both the central and local governments were involved in administering NSP contracts. Several aspects of the relationship between the central and lower administrative regions can affect contract management - the lack of clear delineation in the roles and responsibilities of different administrative levels, inadequate capacity of local governments to manage contracts (e.g. in Bangladesh), and divorcing the financial and monitoring roles (e.g. Tanzania). Finally, in several of the countries studied, donors played an important role in facilitating and financing NSP contracting. In such contexts, good coordination between donors and government is important. In particular, the relationship between NSPs and government should be immune to the vagaries of donor support. For instance the Bangladesh study reported on how withdrawal of donor funding for supporting NSP contracting resulted in changes in the package of services delivered [13].

Government stewardship capacity over contractual arrangements is vital for the success of contracting. In the absence of this, even the best designed programs and contracts will fail to have the desired impact on health outcomes. The case of Bosnia is illustrative of this, where the adoption of supposedly mandatory quality and safety standards has de facto become something of an option, with private providers effectively choosing whether or not to adhere to these standards based on their own assessment of the costs and benefits [17]. This has also been seen in Afghanistan, Tanzania and Bangladesh, where studies have cited political interference in areas such as selection of facilities for contracting and human resource decisions as detrimental to overall project aims [12–14].

Contracted NSPs operate in complex health system environments which can lead to unintended consequences of the contract terms. In Bangladesh, there was an exodus of NSP staff in response to increases in public sector pay scales because the contracts allowed little flexibility for changing salaries. Another example from the Bangladesh study was that of awarding the contract for services to the lowest bidder who cleared a technical screening rather than basing the award itself on a mix of technical and cost criteria. This led NGOs to bid as low as possible, something that had an adverse effect on service quality since providing quality services would put the NGO at a financial loss.

Contracting NSPs is an important policy option for many LMICS seeking to expand and strengthen coverage of publicly financed health services. The studies reviewed in this paper demonstrate the ways in which a diverse set of countries have engaged NSPs to move towards universal health coverage. Contracting NSPs, however, is not without its challenges. If not managed well, it can suffer from many of the resource and management challenges that public sector services face. For countries that have a sizeable NSP sector, making full use of the available human resources by contracting NSPs and appropriately managing them, offers an important way for expanding coverage of publicly financed health services towards achieving universal health coverage.

## Abbreviations

CHAG: Christian Health Association of Ghana
FBO: Faith-Based Organisations
GPCI: General Practitioner Contracting Initiative
IDRC: International Development Research Centre
LMIC: Low and Middle-Income Country
NGO: Non-Governmental Organization
NSP: Non-State Provider
